# Microhabitat and Pollinator Differentiation Drive Reproductive Isolation between Two Sympatric *Salvia* Species (Lamiaceae)

**DOI:** 10.3390/plants11182423

**Published:** 2022-09-16

**Authors:** Tial C. Ling, Patcharin Phokasem, Chainarong Sinpoo, Yong-Ping Yang, Terd Disayathanoowat

**Affiliations:** 1 Bee Protection Laboratory, Department of Biology, Faculty of Science, Chiang Mai University, Chiang Mai 50200, Thailand; 2 Key Laboratory for Plant Diversity and Biogeography of East Asia, Kunming Institute of Botany, Chinese Academy of Sciences, Kunming 650201, China; 3 University of Chinese Academy of Sciences, Beijing 100049, China; 4 Institute of Tibetan Plateau Research at Kunming, Kunming Institute of Botany, Chinese Academy of Sciences, Kunming 650201, China

**Keywords:** pollinator, microhabitat differentiation, floral isolation, reproductive isolation, pre- and post-pollination barriers, sympatric *Salvia* species

## Abstract

Evaluation of multiple barriers contributing to reproductive isolation between sympatric plant species is key to understanding the mechanism of their coexistence; however, such investigations in biodiversity hotspots are still rare. In this study, we investigated and compared geography, microhabitat, phenology, flora, and pollinators, in addition to pollen–pistil interactions, seed production, and seed germination of the closely related sympatric *Salvia digitaloides* and *S. flava* on Yulong Snow Mountain, Southwestern Yunnan, China. The geographic distribution of these species overlapped, but their adaptation to physical and chemical properties of soil microhabitats differed. They shared the same flowering time but differed in flower size, style length, nectar volume, sugar concentration, and flower longevity. Both species shared bumblebees as effective pollinators, but flower constancy for the two species was relatively strong. Pollen tube growth, seed production, and seed germination were lower in interspecific than in intraspecific crosses. Our study suggested that microhabitat and pollinator isolation acted as the most important isolating barriers in maintaining the coexistence of the two *Salvia* species. Our study also highlighted that post-pollination barriers play an important role in preventing the gene flow between these two *Salvia* species.

## 1. Introduction

Speciation can be viewed as a fundamental process of biodiversity that is determined by the evolution of reproductive isolation between previously interbreeding populations [1,2,3,4,5]. The emergence of new species and their maintenance depends largely on a wide range of reproductive barriers [6,7,8,9,10]. The process of speciation is constrained by the barriers contributing to reproductive isolation and how natural selection acts on the formation of reproductively isolated populations from different populations to discrete species [11,12]. The evolution of genetically distinct lineages maintained by reproductive isolation due to geographic or ecological barriers has been studied extensively [1,4,13,14,15,16].

Reproductive barriers can be broadly classified as pre-zygotic and post-zygotic barriers according to the timing of their occurrence [17]. In flowering species, pre-zygotic barriers include pre-pollination barriers of geographic, microhabitat, temporal, floral, and pollinator isolation [18,19]. In contrast, post-zygotic barriers include post-pollination barriers of interspecific incompatibility, embryonic hybrid inviability, and F_1_ sterility [20]. Reproductive barriers can be regarded as forming sequentially: if a pair of species proceeds from the initial stages of divergence toward total isolation, it is expected that multiple reproductive barriers would emerge [11,21]. Therefore, pre-zygotic barriers are the critical initial filter against gene exchange between species and greatly contribute to total isolation in plants in general [22]. In terms of specific mechanisms, broad geographic isolation could act as an initial barrier that contributes toward total reproductive isolation. However, microhabitats, differing in a number of physical and chemical properties, could serve the same purpose for sympatric populations. Changes in physical and chemical properties of soil microhabitats, such as light, water and mineral contents, pH, organic matter, and chemical compounds, could act as the initial barriers due to their effects on both plant and microbial growth [23,24]. Each distinct microhabitat provides a functionally different setting within which closely related sympatric species may respond differently [25]. Although adaption to different microhabitats is regarded as an important driver of speciation, the magnitude of that contribution is little known—particularly with regard to soil’s physical and chemical components. It is still a matter of controversy among evolutionary ecologists whether the maintenance of coexisting species is mostly determined by microhabitat isolation.

When coexisting species occupy similar habitats, floral isolation could limit gene exchange between species. Traits that may drive reproductive isolation include flowering time, flower color, floral scent, nectar characteristics, flower size, and spur length and orientation. Because flower characteristics are mainly selected by pollinators, changes in floral traits often result in pollinator shifts, which can, in turn, induce reproductive isolation. Thus, floral traits greatly impact the degree to which sympatric plants exhibit complete pollinator isolation—even when they share the same flowering period [21,26,27,28,29]. Therefore, studies on the importance of floral isolation and the resultant pollinator partitioning may allow us to understand the mechanisms of plant coexistence and of sympatric speciation. In some cases, sympatric plants share the same generalist pollinators [30], and thereby have the opportunity to exchange genes. In such an instance, post-pollination reproductive isolation may play a role in species divergence: pollen–pistil rejection, inviability of interspecific seed production, or poor germination can cause reproductive isolation between species [30]. Yet more empirical studies of isolating mechanisms generally emphasize one or a few barriers to gene flow, and a few studies have systematically examined the contribution of all potential mechanisms to the total isolation of sympatric species pairs [12,21,31].

**Figure 1:**
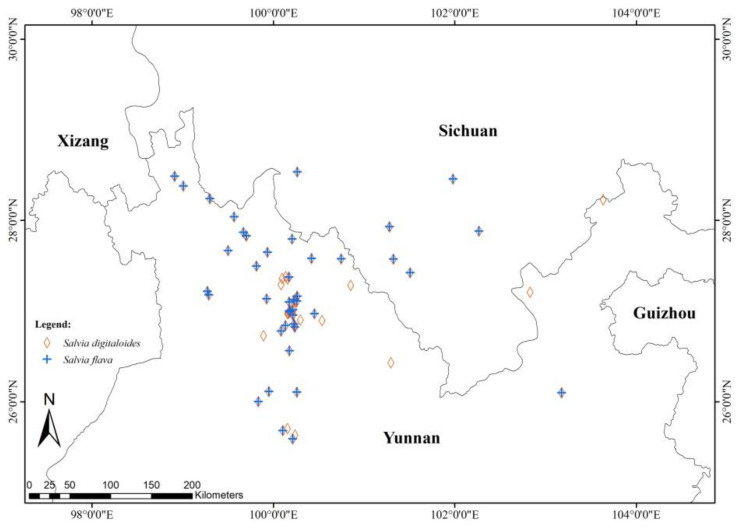
Geographic location of *Salvia digitaloides* (coral diamond, n = 40) and the *S*. *flava* (crosshair blue, n = 59) in Yunnan and Sichuan provinces of China. Geographic distribution ranges of the two species are based on localities in herbarium specimen records in the Chinese Virtual Herbarium, the Chinese National Specimen Information Infrastructure, and the website Global Biodiversity Information Facility.

*Salvia* is the largest genus in the plant family Lamiaceae. It is distributed broadly from the northern to southern hemisphere [32,33]. Flowers of this genus are zygomorphic and offer nectar at the base of the flowers, which are mostly visited by pollinated bees and moths [34]. Many *Salvia* species are reported to co-occur and co-bloom [35,36,37], but reproductive isolation in sympatric *Salvia* species has been investigated in only a few cases [32,38]. Grant [31] documents that reproductive isolation between sympatric *S*. *mellifera* and *S*. *apiana* is mediated by mechanical isolation due to plant adaptation to different pollinators with different body sizes and shapes (also see [37]). The two species are herbaceous perennial shrubs native to the highlands of Southwestern China and have a similar narrow distribution in this region, where most of the populations occur in the Northern Yunnan and Southern Sichuan provinces (Figure 1). In field expeditions, individuals of *S*. *digitaloides* Diels and *S*. *flava* Forrest ex Diels were found to coexist in several areas. *Salvia digitaloides* has flowers with yellowish white petals dusted with a few light purple spots on the lower lip while *S*. *flava* produces flowers with very deep yellow to brownish-yellow petals with a maroon color on the lower lip. The distance between individuals of the two species is approximately 10–50 m at the study sites. *S*. *digitaloides* mostly grows in drier soils and *S*. *flava* grows in muddy areas. Based on the occurrence of the two species in different soil types, soil microhabitats seem to play an important role in contributing to the reproductive isolation between the two species. In addition, pollinator isolation may occur between them if their floral traits (e.g., color and size) attract different suites of pollinators. Flowers of *S*. *digitaloides* are reported to be pollinated by a wide variety of bee pollinators [39]. However, no study has recorded the breeding system and the pollinators of *S*. *flava*. Therefore, empirical studies are needed to test whether soil microhabitats and pollinators are important pre-zygotic barriers contributing to the reproductive isolation between the two species. Likewise, the breeding system and the strength of different stages of post-zygotic barriers that might be important in contributing to reproductive isolation between the two species are rarely studied. In this study, we evaluated a series of potential barriers between the two species and addressed the following three questions: (1) Do closely related sympatric *S*. *digitaloides* and *S*. *flava* require the same soil physical and chemical properties to thrive? (2) Is there reproductive isolation between the two species? (3) If so, do pre-zygotic and post-zygotic barriers have equal contributions to reproductive isolation? We predicted that microhabitat differentiation, floral trait differences, and pollinator isolation contribute mostly to reproductive isolation in the two *Salvia* species.

## 2. Results

### 2.1. Geographic Distribution

We found that the distribution of *Salvia digitaloides* and *S*. *flava* overlapped broadly in the Yunnan and Sichuan provinces of China (Figure 1).

### 2.2. Microhabitat Isolation

#### 2.2.1. Soil Properties

**Figure 2:**
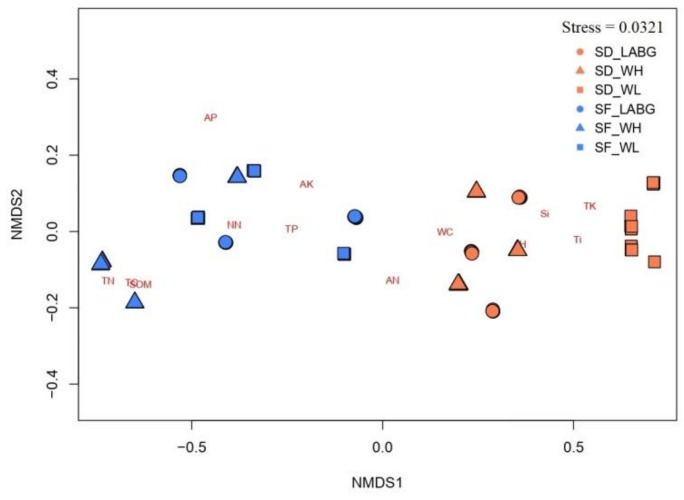
Non-metric multidimensional scaling (NMDS) ordination plot of physical and chemical properties of soil samples collected near flowering individuals of *S*. *digitaloides* and *S*. *flava*. Circle, triangle, and square symbols with coral color denoted Lijiang Alpine Botanical Garden (LABG), Wenhai Village (WV), and Wenhai Lake (WL) study sites of *S*. *digitaloides*. Circle, triangle, and square symbols with blue color denoted LABG, WV, and WL study sites of *S*. *flava*. The measured soil properties were soil water content (WC), pH, soil organic matter (SOM), total nitrogen (TN), nitrate-nitrogen (NN), ammonium nitrogen (AN), total phosphorus (TP), available phosphorus (AP), total potassium (TK), available potassium (AK), silicon (Si), total carbon (TC), and titanium (Ti). Permutational multivariate analysis of variance (PERMANOVA) was used to compare soil properties between the two species at different sites.

There was a significant difference in soil properties between species (R^2^ = 0.71, df = 1, *p* < 0.001) and between sites (R^2^ = 0.10, df = 2, *p* < 0.001), although their interaction was significant (R^2^ = 0.06, df = 2, *p* < 0.001). TK, pH, Si, and Ti were more common in the locations of *S*. *digitaloides*, whereas SOM, TN, NN, TP, AP, AK, and TC were more common in the locations of *S*. *flava* (Figure 2). There was no significant difference between species in WC (based on dry sample) and AN. TK and pH were more common in LABG, whereas Ti, SOM, AK, TC, and WC were more common in the site near WV, and NN, AP, and AN in the site near WL (Figure 2). The properties of Si, TN, and TP equally occurred in all study sites (Table 1).

#### 2.2.2. Spatial Distribution

The mean distance to the nearest conspecific neighbor was 8.33 ± 0.69 m (n = 30) for *S*. *digitaloides* and 5.27 ± 0.37 m (n = 30) for *S*. *flava*. The distance between the individuals of the two species was 20–100 m from each other. Based on 50 × 50 m quadrats, spatial distribution provided a high degree of reproductive isolation (Table 2). The average value of reproductive isolation due to the microhabitat was 0.91 for *S*. *digitaloides* and 0.89 for *S*. *flava*. The value of asymmetry in this barrier was 0.02.

### 2.3. Floral Traits and Longevity

Among measured floral traits, inflorescence number per plant, flower number per plant, corolla tube length, style length, opening width, lower lever’s arm height, flower longevity, nectar volume, and sugar concentration were greater in *S. digitaloides* than those of *S. flava* (Table 3). Stigma lever length, the distance between the stigma and the landing platform, and the distance between the stigma and the lever (horizontal) were greater in *S. flava* than in *S*. *digitaloides* (Table 3). There were no significant differences in corolla tube width, opening length, length of exerted stigma, pollen number, or pollen/ovule ratio between the two *Salvia* species.

### 2.4. Phenological Isolation

**Figure 3:**
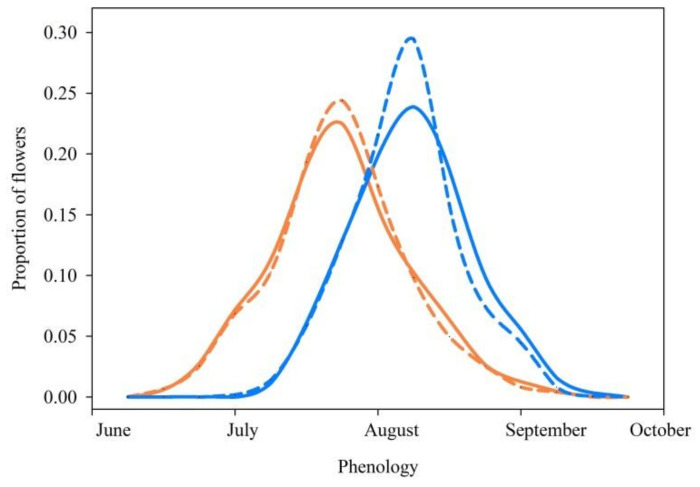
Flowering phenology of *Salvia digitaloides* (coral lines) and *S*. *flava* (blue lines). The solid and dashed lines denote the years of 2015 and 2016 for each *Salvia* species.

In 2015, *S*. *digitaloides* flowered from the 21 June to the 15 September with the peak flowering season on the 26 July, while *S*. *flava* bloomed from the 12 July to the 22 September with the peak blooming season on the 9 August. Thus, the flowering phenology of *S*. *digitaloides* was approximately three weeks earlier than that of *S*. *flava* (Figure 3). The shared flowering period of both species was 77 days. There were 21 days of the unshared flowering period for *S*. *digitaloides* and 7 days for *S*. *flava*. Compared to the flowering time in 2015, the flowering time in 2016 was a week earlier for both species. RI_phenology_ for *S*. *digitaloides* as the female parent was 0.29, whereas RI_phenology_ for *S. flava* as the female parent was 0.21. Thus, the value of asymmetry in this barrier was 0.08.

### 2.5. Pollinator Observation in Natural Populations

**Figure 4:**
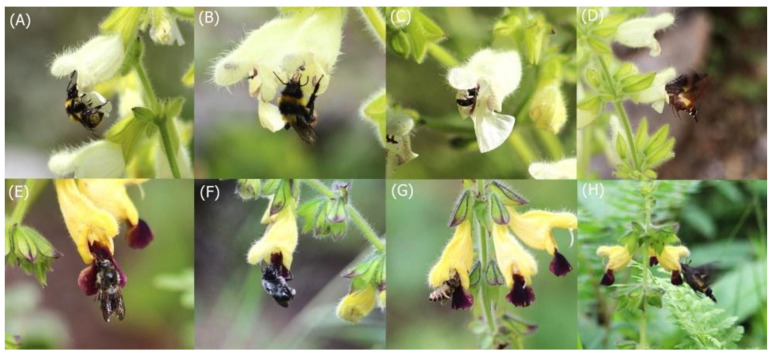
Flowering individuals of *Salvia digitaloides* (from (**A**–**D**)) and *S*. *flava* (from (**E**–**H**)) and their pollinators. *Bombus secures* (**A**), *B*. *friseanus* (**B**), *Dufourea carbopila* (**C**), and *Macroglossum pyrrhosticta* (**D**) visiting flowers of *S*. *digitaloides*. *B*. *remotus* (**E**), *B*. *funararius* (**F**), *Apis cerana* (**G**), and *Macroglossum nycteris* (**H**) visiting flowers of *S*. *flava*.

When pollinators were observed in natural populations, we recorded a total of 165 (68.46%) individuals from four *Bombus* species (*B*. *funararius*, *B*. *friseanus*, *B*. *secures*, and *B*. *remotus*), 45 (18.26%) individuals from *Dufourea carbopila*, 30 (12.03%) individuals from two *Macroglossum* species (*M*. *pyrrhosticta* and *M*. *nycteris*), and 3 (1.24%) individuals of *Apis cerana* visiting the flowers of both *Salvia* species. *B*. *secures* (Figure 4A) and *D. carbopila* (Figure 4C) were found only in *S*. *digitaloides*, whereas *B*. *remotus* (Figure 4E), *B*. *funararius* (Figure 4F), and *A*. *cerana* (Figure 4G) were recorded only in *S*. *flava*. *B*. *friseanus* (Figure 4B), *M. pyrrhosticta* (Figure 4D), and *M*. *nycteris* (Figure 4H) were found visiting both *Salvia* species. We also observed a single visit of *Lasioglossum* species to *S*. *digitaloides* These bees carried hundreds of pollen grains on their proboscides.

*Bombus* species that foraged on the flowers for nectar and pollen received dorsal depositions of the pollen on their heads, thoraces, and infrequently on their abdomens. *Macroglossum* species foraging for the nectar obtained dorsal depositions of the pollen on their heads and thoraces. *D. carbopila* (Figure 4C), *A. cerena* (Figure 4G), and *Lasioglossum* species obtained ventral depositions of pollen on their abdomens, and a few on their heads, wings, and thoraces. *A*. *cerena* foraged for nectar, whereas *D*. *carbopila* foraged for pollen. In 95% (n = 150 flowers for each species) of visits by *B*. *friseanus*, theydid not touch reproductive parts of the flowers when they robed the nectar for either *S*. *digitaloides* or *S*. *flava*.

The mean body size of *Bombus* species visiting *S*. *digitaloides* flowers was significantly larger than the body size of those visiting *S*. *flava* (body size = 46 mm; n = 44 for *S*. *digitaloides* and body size = 43 mm; n = 63 for *S*. *flava*; Kruskal–Wallis test, χ^2^ = 5.253 df = 1, *p* < 0.05). There were no significant differences in thorax width (56 mm for *S*. *digitaloides* and 52 mm for *S*. *flava*; χ^2^ = 0.446, df = 1, *p* = 0.506) or thorax depth (55 mm for *S*. *digitaloides* and 52 mm for *S*. *flava*; χ^2^ = 0.631, df = 1, *p* = 0.427) between *Bombus* visitors of the two *Salvia* species.

### 2.6. Pollinator Isolation in Controlled Choice Experiment

In a controlled choice experiment containing potted plants of both *Salvia* species, we recorded 155 individuals of *Bombus* species, 16 individuals of *Macroglossum* species, and 14 individuals of *D*. *carbopila* that foraged on the flowers of both *Salvia* species in the daytime. This resulted in 1306 intraspecific (518 *S*. *digitaloides* → *S*. *digitaloides*, and 788 *S. flava* → *S. flava*) and 102 interspecific transitions (24 *S*. *digitaloides* → *S*. *flava*, and 78 *S*. *flava* → *S*. *digitaloides*) for *Bombus* species; 120 intraspecific (67 for *S*. *digitaloides* → *S*. *digitaloides*, 53 for *S*. *flava* → *S*. *flava*) and 1 interspecific transitions (*S*. *flava* → *S*. *digitaloides*) for *Macroglossum* species; and 36 intraspecific (*S. digitaloides* → *S*. *digitaloides*) and 1 interspecific transition (*S*. *flava* → *S*. *digitaloides*) for *D. carbopila*. In total, 90.38% of interspecific transition was obtained from *B*. *friseanus*, 6.33% from *B*. *funararius*, and 0.96% of interspecific transition from each of *D. carbopila*, *B*. *secure,* and *Macroglossum,* respectively. *B*. *remotus* showed absolute floral constancy. Values of the degree of reproductive isolation, floral constancy, and asymmetry in the selective foraging barrier per pollinator for *S*. *digitaloides* and *S*. *flava* are shown in Table 4. No pollinator was found visiting the flowers of the two *Salvia* species at night.

### 2.7. Pollen–Pistil Interactions

Based on the examination of the placement of pollen grains on a stigma for 48 h, the number of pollen tubes penetrating ovaries varied among treatments for each species (χ^2^ = 23.97, df = 2, *p* < 0.001 for *S*. *digitaloides* and χ^2^ = 9.246, df = 2, *p* < 0.05 for *S*. *flava*). Both self (mean = 66, n = 27) and intraspecific (75, n = 30) pollinations of *S*. *digitaloides* flowers had an equal number of pollen tubes penetrating the ovaries; and they were significantly more than number of pollen tubes in the ovaries of interspecific pollinated flowers (34, n = 25; *p* < 0.001). In *S*. *flava*, self (62, n = 29) and intraspecific (76, n = 30) pollinated flowers had an equal number of pollen tubes entering the ovaries. Interspecific (51, n = 30) pollination in *S*. *flava*, as the female parent, still obtained as many pollens as that of self-pollination; however, it was significantly lower than the pollen number of intraspecific pollination (*p* < 0.05). Reproductive isolation due to pollen–pistil interaction was 0.38 for *S*. *digitaloides* as the female parent and 0.19 for *S*. *flava* as the female parent. The asymmetric value in this barrier was 0.19.

### 2.8. Seed Production

**Figure 5:**
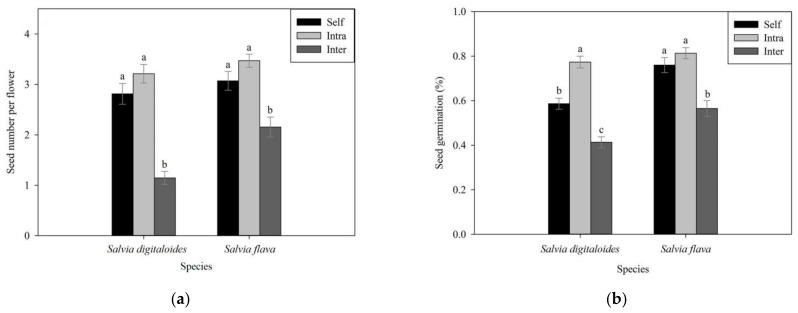
(**a**) Seed production and (**b**) germination of hand self- (black bars), intra- (light gray bars), and interspecific (dark gray bars) pollinations for *Salvia digitaloides* and *S*. *flava*. Different letters indicate significant differences at *p* < 0.05. Error bars represent mean ± standard errors.

The bagged inflorescences which flowers were aimed to test for the capacity of autonomous selfing did not set any seed. Seed production varied between treatments (χ^2^ = 115.08, df = 5, *p* < 0.001) and between species (χ^2^ = 12.75, df = 1, *p* < 0.001), and the interaction was also significant (χ^2^ = 115.08, df = 5, *p* < 0.001). In both species, the seed number of hand self-pollinated flowers (2 (n = 43) for *S*. *digitaloides* and 3 (n = 42) for *S*. *flava*) and intraspecific cross-pollinated flowers (3 (n = 47) for *S*. *digitaloides* and 3 (n = 51) for *S*. *flava*) species produced an equal number of seeds, and it was significantly greater than the seed numbers of interspecific cross-pollinated flowers (1 (n = 62) for *S*. *digitaloides* and 2 (n = 58) for *S*. *flava*; Figure 5a). Seed numbers of interspecific cross-pollinated flowers in *S*. *digitaloides* as the female parents was significantly fewer than seed numbers of that in *S*. *flava* as the female parents. Interspecific isolation in *S*. *digitaloides* and *S*. *flava* as female parents for seed production was 0.50 and 0.23, respectively. The asymmetric value in this barrier was 0.27.

### 2.9. Seed Germination

There was a significant variation in the proportion of seed germination between treatments (χ^2^ = 28.807, df = 2, *p* < 0.001), and between species (χ^2^ = 7.137, df = 1, *p* < 0.01), although their interaction was not significant (χ^2^ = 1.109, df = 2, *p* = 0.574). Compared to the seed germination of self-pollinated seeds (59% (n = 75) for *S*. *digitaloides* and 76% (n = 75) for *S*. *flava*) and intraspecific cross-pollinated seeds (77% (n = 75) for *S*. *digitaloides* and 0.8% (n = 75) for *S*. *flava*), the germinated proportion of the seed in interspecific cross-pollinated was much lower in both species (0.4% (n = 75) for *S*. *digitaloides* and 0.6% (n = 75) for *S*. *flava*; Figure 5b). The proportion of seed germination in self-pollinated seeds was still lower than that in intraspecific cross-pollinated seeds in *S*. *digitaloides* (Figure 5b). There was no significant difference in the proportion of seed germination between intra- and inter-specific cross-pollinated seeds for *S*. *flava*. Reproductive isolation due to seed germination for *S*. *digitaloides* and *S*. *flava* as female parents was 0.31 and 0.18, respectively. The value of asymmetry in this barrier was 0.15.

### 2.10. Total Reproductive Isolation

**Figure 6:**
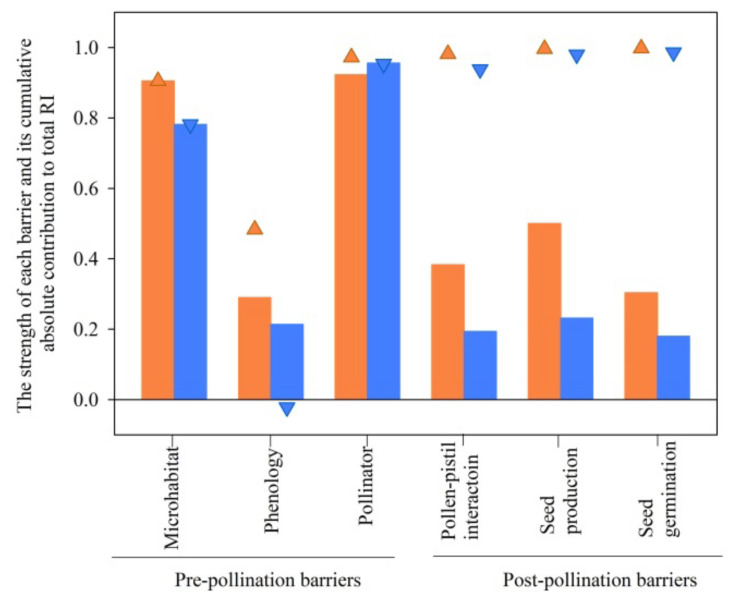
The degree of six barriers (bars) and their accumulative absolute contributions (triangle symbols) to total isolation in reciprocal crosses of the two species. Bars and triangle up symbols with coral color represent *S*. *digitaloides*, while bars and triangle down symbols with blue color represent *S*. *flava*.

The total reproductive isolation was 0.98 for *S*. *digitaloides* and 0.97 for *S*. *flava* as female parents. The relative contribution of each individual barrier to the total reproductive isolation ranged from −0.83 to 0.96. The higher contribution was detected at the stage of pollinator isolation and followed by microhabitat isolation (Figure 6).

## 3. Discussion

### 3.1. Ecogeographic Isolation

Based on data from herbarium records and personal observations, the geographic distribution of the Chinese endemics *S. digitaloides* and *S*. *flava* are limited to the Yunnan and Sichuan provinces of China. The two species share the same large-scale geographic distribution. Our result mirror reports of closely related sympatric taxa in the Hengduan Mountain biodiversity hotspot, such as pink and white morphs of *Spiranthes sinensis* [12], *Herbanaria* species [25], *Pedicularis* species [30], and yellow and purple morphs of *Roscoea cautleoides* (unpublished data).

Because geographic distribution is a relatively weak barrier, microhabitat isolation due to spatial distribution acts as the initial barrier to gene flow between the two *Salvia* species. The contribution of spatial distribution to the total reproductive isolation between the two species was 90.1 for *S. digitaloides* and 88.5 for *S*. *flava*, indicating a strong spatial distribution between the two species. This result was based on 50 × 50 m quadrat observations at the focal sites. The strength of spatial isolation between the two species could be drastically weak if we increased the quadrat size. In other words, the potential for the share in spatial distribution between the two species could increase as the sampling/quadrat size increase. In this study, the distance between individuals of the two species ranged from 10 to 50 m as mentioned in the methods (see Section 4.7). Our aim was to estimate microhabitat isolation due to spatial distribution and soil microhabitats at small scales. Thus, our result still reflected some degrees of microhabitat isolation caused by spatial distribution at small scales. Future studies of microhabitat isolation in sympatric species may include a broader range of species to determine if spatial distribution plays a role in maintaining species boundaries. 

We found that most of the physical and chemical properties obtained from soil microhabitats differed between the two *Salvia* species. These results support the finding of a previous study on *Spiranthes sinensis*, where soil water content varied among the individuals with different colors in this focal region [25], also see [40]. Therefore, changes in soil properties and chemical components seem to be important mechanisms that possibly cause plant adaptation to different soil microhabitats in this region. In other words, the two *Salvia* species may have different soil requirements, resulting in an adaptation to different microhabitats within the same regions. However, we cannot determine whether microhabitat isolation between the two species is solely due to these chemical properties of the soil without further study of more populations and environmental factors, such as humidity, light intensity, temperate, and stress in both manipulated laboratory transplants and in the natural populations. In general, the nectar and pollen foraging distance of some bees (e.g., *Bombus* species) can be up to several kilometers from their colonies [41,42], and pollen remained about 5% viable after 150 min [43]. Therefore, gene flow between the two species could occur if they flower at the same time and share the same pollinators. Nevertheless, based on the present results, microhabitat isolation may play an important role in phenotypic isolation, and not pollination isolation, as such microhabitats are within the foraging distance of pollinators.

### 3.2. Phenology Isolation

Variations in flowering phenology among co-existing plant species can be viewed as niche partitioning mechanism that promotes species coexistence and diversity. Like other findings from the same focal study sites [12,25,30], we found that the two species bloomed in the same period from June to July, suggesting that phenology isolation due to the flowering time is relatively weak. Our findings are inconsistent with sympatric *S*. *apiana* and *S*. *mellifera* which bloomed at different times of the year in North America [37]. A few studies have suggested that flowering time can vary among sympatric species or among populations within the same species at both the population and community levels [11,25,37]. Conversely, a recent study suggested that populations of *H*. *limprichtii* and *H*. *davidii* in the Hengduan Mountain in the northern region of Yunnan shared flowering times [25]. In the present study, although the three populations had slight differences in the slop orientation, we did not find any difference in flowering time among the populations or years. This could be due to restricted population sizes located within the same elevation ranges. Future studies may focus on flowering phenology of the two species at a large scale. Nevertheless, the current results suggest that the strength of phenology isolation between the two species is relatively low. Thus, there could be opportunities for interspecific pollination unless other mechanisms (e.g., flower–pollinator interactions) restrict the gene flow.

### 3.3. Floral Trait Isolation and Flower Constancy

Because some flower characteristics are mainly selected by pollinators, changes in floral traits often result in pollinator shifts, which could induce the strength of reproductive isolation between sympatric species. Variations in flower size, spur length, and scent compounds among orchid species [25,44,45], the difference in the nectar composition and the corolla length of *Roscoea* species [11,46], and the color variations in the individuals of *Spiranthes sinensis* [12] have been reported to attract different pollinators, and in turn promote reproductive isolation. Consistent with these trends, our study showed that floral traits (e.g., size, color, nectar contents, and pollen production) differed between the two *Salvia* species. Although we did not examine the difference in the components of floral volatile compounds between the two species, we could easily detect that the odor of *S*. *digitaloides* was very strong while *S*. *flava* was very mild.

In this study, we did not measure the mechanical isolation of *Salvia* species; however, their floral morphometrics partly exhibited some degrees of mechanical isolation. The stigmas of the two *Salvia* species contacted the dorsal surfaces of *Bombus* and *Macroglossum* species. In contrast, *Dufourea carobopila* and *Apis cerana* inverted their bodies while foraging on the flowers. Specifically, *D*. *carobopila* always collected pollens through ventral surfaces of their thoraces and rarely touched the stigmas. The mechanical isolation may be incomplete in these studied plant species because all *Bombus*, which were the most frequent interspecific visitors, shared the same foraging behaviors. However, it is important to know that the body size of *B*. *friseanus* did not always fit to the natural opening size of the flowers, and it was pronounced in the flowers of *S*. *flava*. As a result, they pierced holes into the flowers of *S*. *flava* for the nectar and failed to contact the reproductive parts of the flowers. These patterns show the nectar robbing mechanism in *Salvia* species, and these have been found in many species [47,48,49]. For example, Ye et al. [50] suggested that both nectar robbing and pollinator visitation was influenced by the floral diversity of *S*. *przewalskii*.

On the other hand, our studies showed a high degree of flower constancy due to their selective foraging on flowers of the two *Salvia* species, indicating a strong contribution of pollinators to total reproductive isolation. Similar results have been reported in several sympatric species, where flowers attract different pollinators [11,21]. Although *B*. *friseanus* often made interspecific visits, the difference in mechanical isolation due to the mismatch between the body size of bumble bees and the opening size of flowers may restrict interspecific pollen exchanges. Providing the ability of pollinators in pollen deposition may allow us to confirm the strength of mechanical isolation. In addition, observing pollinators in controlled experiment choices with different spatial separations between the two species under many environmental conditions could also allow us to confirm their contribution to reproductive isolation.

### 3.4. Post–Pollination Isolation

Pollen–pistil interactions are the first stage of post-pollination isolation, and therefore are crucial to prevent gene flow between the sympatric plant species [25,29,30,44]. In this study, we found a significantly lower number of interspecific pollen tubes penetrating the stigmatic surface and the ovary of *S*. *digitaloides*, but not *S*. *flava*. This is in contrast to the occurrence of strong mechanical isolation between *S*. *apiana* and *S*. *mellifera* [51]. Perhaps, this is related to the style length differences between the two species. Tong and Huang [52] examined pollen–pistil interactions of interspecific crosses after 24 h and found that the pollens from short-style *Pedicularis* species could not penetrate the entire length of long-style species, leading to unilateral pollen–pistil isolation. In addition, Liang et al. [53] suggested that although three sympatric *Pedicularis*, with similar style lengths, did not manifest any correlation between style length and pollen tube growth after 24 h of interspecific crosses, they still grew until they reached 48 h. In this study, the style length of *S*. *digitaloides* was much longer than that of *S*. *flava*, suggesting the potential restriction of *S*. *flava* pollen tube growth in *S*. *digitaloides* pollen. Future studies could investigate pollen–pistil interactions in the two species over a longer time span as the flowers of both species last 4 to 5 days.

Bagged and intact flowers did not set any seed, indicating that both *Salvia* species succeed in pollination only through pollinators. Post-pollination barriers often show strong reproductive isolation between closely related species [21,53]. For example, Cuevas et al. [35] suggested that the reduction in seed production and seed germination are likely the main barriers preventing the formation and the establishment of hybrids between *S*. *elegans* and *S*. *fulgens*. In contrast, manual interspecific crosses of *S*. *digitaloides* and *S*. *flava* produced a relatively low seed set and seed germination, suggesting strong post-zygotic isolation between the two species. However, many flowers from interspecific crosses still produced seeds and successfully germinated. This implies that hybridization between the two species is possible if pollinators make an interspecific visit. Unfortunately, we did not evaluate the seedling growth of hybrid seeds; therefore, we still do not know whether the additional post-zygotic barriers play a role in isolation. Based on microhabitat isolation, the survival rate of hybrid seedlings in natural populations is likely to be restricted unless other mechanisms enhance their survival rate.

### 3.5. Contribution of Pre- and Post-Pollination Isolation to Total Isolation

Several studies have documented that the contribution of pre-pollination barriers to total isolation is stronger than that of post-zygotic barriers [10,11,21,53]. Among pre-zygotic barriers, microhabitat isolation and pollinator isolation were the main mechanisms contributing to the total reproductive isolation in these two species. All *Salvia* species showed strong pre-zygotic isolation, with *S*. *apiana*/*S*. *mellifera* and *S*. *elegans*/*S*. *fulgens* [35,45] demonstrating other patterns. Post-zygotic reproductive isolation between the *S*. *digitaloides* and *S*. *flava* was strong but not absolute. Their total reproductive isolation depends on other post-pollination barriers, such as hybrid seedling survival, growth rate, and productivity. Nevertheless, some studies have argued that the existence of a complete pre-zygotic isolating barrier can effectively block hybridization and therefore maintain the sympatric species, e.g., [30]. More studies on both pre- and post-zygotic isolating barriers are needed to fully understand the mechanisms of species coexistence and diversity in *Salvia*, being important to explore more species pairs with different phylogenetic relationships

## 4. Materials and Methods

### 4.1. Study Species and Sites

*Salvia digitaloides* and *S*. *flava* species, native to Southwestern China [54], are perennial herbs and distributed mainly at altitudes ranging from 2000 to 3900 m in the Northwestern Yunnan and sSuthwestern Sichuan provinces of China (Figure 1). The two species have similar vegetative appearances and floral shapes; however, they can be easily distinguished by their flowers (Figure 4). The flowers of *S*. *digitaloides* have yellowish white petals with a dusting of a few light purple spots on the lower lip of the bilabiate corolla, whereas the flowers of *S*. *flava* have very deep yellow to brownish-yellow petals with a maroon color on the lower lip of the bilabiate corolla. The flowers of these species are bisexual, protandrous, nectariferous, and zygomorphic in shape, with a hooded upper lip. The style of the flowers exerts out of the upper lip and the stigma is far away from the anthers. Two stamens are modified as the staminal lever, with the upper theca of each stamen fertile and the lower one reduced [39,55]. The flowers of both species have four ovules. *S*. *digitaloides* flowers are mostly visited by bumblebees [32,39]. The latest published phylogeny of the genus *Salvia* suggested that *S*. *digitaloides* and *S*. *flava* are sister species in clade 6 [56]. In natural populations, *S*. *digitaloides* plants grow mostly in dry shady pine forests or on open grassy hillsides and valleys. In contrast, *S*. *flava* plants are more common on hillsides and along stream banks in wet gravelly soil. Although *S*. *digitaloides* is already known to be reported to be mostly visited by bumble bee pollinators [39], there is no empirical study on the breeding system of this species. Specifically, to the best of our knowledge, there is no information about the mating systems of *S*. *flava*.

We conducted field experiments at Yulong Mountain (27°00′09.44′′ N, 100°10′49.41′′ E, 3282 m asl), Lijiang, Northwestern Yunnan, China. All field experiments were conducted at three natural sites across three different years (2015, 2016, and 2019). The study sites were located at the Lijiang Alpine Botanical Garden field station (LABG: 27°0′1.19′′ N, 100°10′49.25′′ E, 3262 m asl), near Wenhai village (WV: 26°58′45.05′′ N, 100°10′41.21′′ E, 3186 m asl), and near Wenhai Lake (WL: 26°58′32.22′′ N, 100°10′24.25′′ E, 3262 m asl). The distance between the sites ranged from 2.3 to 4.0 km.

### 4.2. Geographic Distribution

To investigate if the two *Salvia* species are sympatric at larger macro-spatial scales, we drew their distribution range map based on a database constructed from online information on specimens. We first downloaded all the herbarium specimen records and locations of both *S*. *digitaloides* and *S*. *flava* from the Chinese Virtual Herbarium (CVH, http://www.cvh.ac.cn; accessed on 2 March 2020), the Chinese National Specimen Information Infrastructure (NSII, http://www.nsii.org.cn; accessed on 3 March 2020), and the website Global Biodiversity Information Facility (GBIF, http://www.gbif.org; accessed on 7 March 2020). We excluded specimens that had been identified incorrectly or had duplicate records. In total, we obtained 83 herbarium specimen records of *Salvia digitaloides* and 143 of *S*. *flava*. After removing duplicate records and unknown location records from each of GBIF, NSII, and CVH, only 40 records of *S*. *digitaloides* and 59 records of *S*. *flava* were left to make a range map using ArcGIS. We then searched the locality on Google Earth to obtain longitude and latitude data to make their distribution range map using ArcGIS 10.2 software.

### 4.3. Microhabitat Isolation

#### 4.3.1. Soil Properties

To investigate microhabitat isolation between the two species, soil cores were established in each of the three studied plots. The cores set at the base of the plant species were made with a sharp stainless-steel drill with a 9 cm inner diameter. After removing the litter layer, the core was dug from 0 to 20 cm depth, homogenized, and passed through a 2 mm mesh sieve. For physicochemical analysis, soil samples were kept in a sterilized self-sealing bag and stored at 4 °C. and then immediately transported to a laboratory for analysis. For soil characteristics assessment, a total of 13 soil physical and chemical properties were examined including soil water content (WC), pH, soil organic matter (SOM), total nitrogen (TN), nitrate-nitrogen (NN), ammonium nitrogen (AN), total phosphorus (TP), available phosphorus (AP), total potassium (TK), available potassium (AK), silicon (Si), total carbon (TC), and titanium (Ti). Soil physical and chemical properties were quantified using standard techniques recommended by a soil scientist at the Central Laboratory of Xishuangbanna Tropical Botanical Garden, Chinese Academy of Sciences. Detailed protocols for measuring soil water content are available [23,57,58,59,60]. Soil pH was measured in a 1:1 soil–water suspension with a pH meter (pHS-2, Shanghai Leici, Shanghai, China). Soil organic matter was determined by the potassium dichromate oxidation method. TN was determined using micro-Kjeldahl digestion followed by steam distillation. NN and AN were determined by steam distillation and indophenol-blue colorimetrically, respectively. TP and AP were quantified using the molybdenum-antimony anti-spectrophotometric method; TK and AK were measured by flame photometry. TC based on dry samples was measured by the LECO carbon analyzer. The concentration of Si was read by using an atomic absorption spectrometer (Agilent AAS-240FS). Ti was extracted by using the Kroll process (magnesium reduction).

#### 4.3.2. Spatial Distribution

In 2016 and 2019, at each site, we examined the extent of the small-scale spatial isolation between the two species by quantifying the degree of co-occurrence. We randomly placed 10 quadrats of 50 × 50 m within a predefined 500 × 500 m plot and counted the number of quadrats containing only *S*. *digitaloides*, only *S*. *flava*, and both species. In 2019, we also recorded the nearest distance between conspecific plants (30 plants for each species) within the quadrats for each species at each study site. For each year, we pooled data from the three study sites and determined the proportion of quadrats that were shared and unshared for each species. From these proportions we calculated microhabitat isolation for each year following the Equation (4C) of Sobel and Chen [61]:

RI_microhabitat_ = 1 − (S/(S + U)) (1)

where S represents shared microhabitat between the two species, whereas U represents unshared microhabitat between them.

### 4.4. Floral Traits and Longevity

In 2015, we recorded the number of inflorescences per plant and the number of flowers per inflorescence from 30 different plants per species. Moreover, we collected 30 freshly opened flowers of each *Salvia* species and measured the following morphological traits: corolla tube length and width, length of exerted stigma, lower lever’s arm height, opening length and width, stigma lever length, style length, the distance between stigma and lever (horizontal), and the distance between stigma and landing platforms. We used digital calipers to measure these traits to a resolution of 0.01 mm. For all trait measurements, we used only one flower from the middle whorl of an inflorescence per plant to prevent repetition and ensure independence.

To determine the total number of pollen grains per flower, we collected one flower from each of 15 plants per species and fixed each flower separately in a Formalin Aceto-Alcohol (FAA) solution (formalin:acetic acid:ethanol at a ratio of 5:5:90 by volume). All anthers in each flower bud were dissected and all pollen grains were collected in a 1.5 mL micro-centrifuge tube with a suspension of 0.5 mL of a mixed FAA solution and detergent. In each observation, 10 subsamples (10 µL each) were placed on a glass microscope slide, and the total number of pollen grains on the slide was counted under a light microscope (XSZ-0900, Wuzhou Oka Optical Instrument Co., Ltd., Wuzhou, Guangxi, China).

To determine nectar volume and sugar concentration, we randomly selected 30 plants for each species and bagged their flowers with fine nylon nets. We bagged the flowers when they were in bud to exclude all insect visitors. On the second day after anthesis, nectar was collected from a single flower from the middle whorl of one inflorescence per plant, using 100 × 0.5 mm glass capillary tubes (Instrument Factory of West China Medical University, Chengdu, Sichuan, China). Collections were timed for peak nectar secretion periods, between 0900 and 1200 h. Nectar volume was determined by measuring the height to which the nectar filled the tube with a digital caliper (0.01 mm precision, Guilin Guangdu Measuring Instrument Co., Ltd., Guilin, Guangxi, China) and the length measurements were then converted to microliters. The sugar concentration was measured using a hand-held, temperature-compensated refractometer (Eclipse; Bellingham and Stanley Ltd., Turnbridge Wells, Kent, UK).

We also determined the flower longevity of an additional 30 flowers for each species by counting the number of days from the bud opening to the day the corolla wilted.

### 4.5. Phenological Isolation

To quantify the synchrony of the flowering period of both *Salvia* species, we conducted a total of 15 phenology censuses for each species in each study site from 14 June to 22 September in 2015 and 2016. Each year, prior to floral phenology observations, we randomly marked 200 healthy plants prior to the flowering of each species. We then recorded the numbers of open flowers on all marked plants every 5 to 7 days, because the average life span of a single flower of *S*. *flava* is 4.83 days and that of *S*. *digitaloides* is 6.71 days. We also recorded the number of days both species shared and unshared the blooming periods. We calculated the degree of phenological isolation for each year as a reproductive barrier following Sobel and Chen [61]:

(2) $${\mathrm{RI}}_{\mathrm{phenology}}=1\;\times \;2\;\frac{\frac{\sum_{i}\left(\frac{A_{i}}{A_{\mathrm{total}}}+\frac{B_{i}}{A_{i}+B_{i}}\right)}{B_{\mathrm{total}}/\left(A_{\mathrm{total}}+B_{\mathrm{total}}\right)}}{\begin{array}{c} \frac{\sum_{i}\left(\frac{A_{i}}{A_{\mathrm{total}}}+\frac{B_{i}}{A_{i}{+B}_{i}}\right)}{B_{\mathrm{total}}/\left(A_{\mathrm{total}}+B_{\mathrm{total}}\right)}+\frac{\sum_{i}\left(\frac{A_{i}}{A_{\mathrm{total}}}+\frac{A_{i}}{A_{i}{+B}_{i}}\right)}{A_{\mathrm{total}}/\left(A_{\mathrm{total}}+B_{\mathrm{total}}\right)} \end{array}}$$

where A*_i_*/A_total_ represents the proportion of species A available for mating on day *i* to its total abundance throughout the flowering season. B*i*/(A*i* + B*i*) represents relative abundance of the heterospecific species on that day, while A*i*/(A*i* + B*i*) represents relative abundance of the interspecific species on day *i*.

### 4.6. Pollinator Observation in Natural Populations

To examine the variation in flower visitation among the pollinators of each *Salvia* species in natural populations, we observed the numbers of each pollinator species visiting flowers of each *Salvia* species in the three studied populations. The observation was conducted on clear sunny days from 08:00 to 18:30 h in 2015 and 2016. We discarded observing the pollinators in situ at night. The observation was performed in July and August, both of which were the peak flowering season of the two *Salvia* species in the study populations. On each observation day, we selected 5 to 10 flowers per inflorescence from 10 different plants and used 5 to 10 inflorescences per plant. We then recorded the number and the foraging behavior of each insect visitor on the individual flowers of the two *Salvia* species. The observations were done for all populations and both species on either the same day or one close to the day, as differences in observation timepoints could affect the visitation rate, depending on the flower visitor phenology. We considered a legitimate pollinator only when it contacted the reproductive parts of the flower, although we noted that this interaction alone did not confirm that a given visitor had a positive effect on pollination. Although all flower visitors were effective pollinators, *Bombus friseanus* often acted as a nectar robber by piercing holes in the flowers when their body sizes did not fit to the opening size of the corollas and failed to touch reproductive parts of the visited flowers. Nevertheless, our aim was to determine the variation in the proportion of flower visitation among the pollinators for each *Salvia* species. Thus, we still included the nectar robbing data in our analyses. We restricted the collections of insects to those observed for foraging bouts. We did an additional specimen collection on *Salvia* flowers in natural populations for identification. Collected specimens were netted and euthanized in jars with fumes of ethyl acetate. Specimens were pinned, labeled, measured, and sent to entomologists for identification (see Acknowledgements). Vouchers were deposited at the Kunming Institute of Botany, Chinese Academy of Sciences, Kunming.

### 4.7. Pollinator Isolation in Controlled Choice Experiment

As the individuals of *S*. *digitaloides* and *S*. *flava* grew about 10–50 m distances from each other at our study sites, we were unable to observe the frequency of insect visitors switching between species during the same foraging bouts. Therefore, we made pollinator observations by transplanting each *Salvia* species in an open meadow field. To avoid the influence of context choice experiences by insects, we chose an open meadow that was isolated from all other flowering sites, particularly where both *Salvia* species and other common/dominant flowering plants (e.g., *Pinus*-*Quercus* forests) grew at least 200 m away. For each species, we dug up 50 healthy plants with flower buds and replanted each on its own plastic pot filled with a little water to keep them fresh. We then covered each inflorescence with green nylon bags tied at its base to prevent from insect disturbance. On sunny days, we transferred those potted plants to an open field and performed controlled choice experiments. For each experiment, flowers were placed in 10 rows of 10 such that adjacent rows were offset by half the distance between flowers in each row, following the methods of Gegear and Thomson [62]. The spatial separation from any flower to each of the near and second-near neighbors was 20 cm. We distributed 100 flowers of each *Salvia* species in alternating rows of two in order to allow flower visitors to have an equal choice of both species upon leaving any flower. We then observed pollinator visitation to flowers of both species from 08:00 to 18:30 h for 30 sunny days in 2015 and 2016. For both species, we additionally observed flower visitors during the night from 20:00 to 22:00 h and from 00:00 to 06:00 h to examine the strength of the flower constancy via nocturnal pollinators. We did 10 days of the observation during the peak flower season of each *Salvia* species in each year. We recorded the number of flowers visited by each pollinator and the transitions and foraging bouts of each pollinator between the flowers of intra- and inter-species. A foraging bout started when a visitor landed on the flower of either *S*. *digitaloides* or *S*. *flava* species until it was lost from view or foraged on flowers belonging to other species. Transitions are either intraspecific (between flowers of the same species, e.g., *S*. *digitaloides* → *S*. *digitaloides* or *S*. *flava* → *S*. *flava*) or interspecific (between flowers of different species, e.g., *S*. *digitaloides* → *S*. *flava* or *S*. *flava* → *S*. *digitaloides*). The pollinator foraging preferences for each species was calculated following the equations of Sobel and Chen [61]:

(3) $${\;\mathrm{RI}}_{\mathrm{pollinator}}=1-2\times \left(\frac{H}{H+C}\right)$$

where C refers to the proportion of intraspecific pollinator foraging, and H refers to the proportion of interspecific pollinator foraging between *S*. *digitaloides* and *S*. *flava*. Based on this experiment of foraging choices, Gegear’s constancy index (CI) was used to calculate the constancy of individual pollinators (Gegear and Thomson) [61]:

(4) $$\mathrm{CI}=\left(c-e\right)/\left(c+e-2ce\right)$$

where *c* is the proportion of intraspecific visits, and *e* is the expected proportion of interspecific visits. If *p* is the proportion of visits to one of the plants, then *e* = *p*^2^ + (1 *− p*)^2^. Possible values ranged from −1 (complete inconstancy) to 0 (random foraging) to 1 (complete constancy).

### 4.8. Pollen–Pistil Interactions

In 2016, we quantified inter-specific pollen–pistil interactions for each *Salvia* species by performing reciprocal interspecific hand-pollinations for both species pairs and intraspecific hand-pollinations within each species. Three flowers from the middle whorl of each of the 30 inflorescences from 30 plants were emasculated before their anthers dehisced and bagged with green nylon mesh bags. On the second day of anthesis, each of the selected flowers was subdivided into three-hand pollination treatments as follows: (1) self-pollination, in which the pollen was removed and deposited on the stigma of the same flower, (2) intraspecific pollination, in which the pollen was removed from one flower and then deposited on the stigma of a flower on a second inflorescence growing at least 10 m away, and (3) interspecific pollination, in which the pollen was removed from flowers of both species and deposited on the stigma of the other species that had its pollen removed. We collected pistils after 48 h, then kept them separately for each treatment per species in 50 mL sterilized glass bottles with 3:1, 95% ethanol: glacial acetic acid for 12 h, and then decanted the preservative, replacing it with 70% ethanol [63]. Upon returning to the laboratory, each specimen was softened and cleared in separate glass vials by submerging each one in a 0.10 g.mL^−1^ solution of sodium sulfite at 45 °C for 2 h. We carefully washed those softened specimens in deionized water. We then excised each pistil by splitting them longitudinally with razor blades and mounting them on different glass slides. The remaining flower was discarded. We counted the number of pollen tubes penetrating the ovary for each pollination treatment of each *Salvia* species. RI of pollen–pistil interaction was calculated using Equation (3) following Sobel and Chen [61] where H refers to the number of interspecific pollen tubes entering the ovary; and C refers to the number of the intraspecific pollen tubes entering the ovary.

### 4.9. Seed Production

To test for the capacity of autonomous selfing, 2–4 flower buds from the middle whorl of each of the 30 inflorescences (one inflorescence per plant) from 30 different plants for each species were covered with a fine nylon mesh bag and never performed hand pollinations. We conducted reciprocal hand pollination experiments following the same protocols as above in 2016. We repeated each treatment with 2–4 flowers of each inflorescence. Approximately a month later, seeds were counted and kept separately in seed-paper bags for each treatment per species. We excluded manipulated flowers that were damaged after emasculation. Reproductive isolation due to seed production was calculated using Equation (3) following Sobel and Chen [61]; here H denotes seed numbers of interspecific cross-pollinations; C denotes seed numbers of intraspecific cross-pollinations.

### 4.10. Seed Germination

We carried out seed germination experiments for each hand-pollination treatment to estimate the post-zygotic isolation. We dried the seeds (separated by treatments) obtained from each of the six pollination treatments at room temperature for two months. We then kept them in paper bags and stored them at 4 °C in a refrigerator before the germination experiments. For seed germination experiments from each pollinated treatment, 75 seeds were evenly separated into five replicates of 15 seeds and placed on wet filter paper in Petri dishes to measure seed germination. The seeds were then placed in an incubator at 20 °C in the Germplasm Bank of Wild Species, Kunming Institute of Botany, Chinese Academy of Sciences. We investigated germination rates every 2 days for 14 days from 1–14 May 2017, while ensuring that they remained moist. The number of seeds that germinated in each Petri dish was counted, and the seed germination rate for each replicate was then calculated by dividing the number of germinated seeds by 15. Then, we calculated the strength of reproductive isolation due to the seed germination rate as a barrier using equation (3) of Sobel and Chen [61], where H denotes the germination rates from interspecific crosses, and C denotes the germination rates from intraspecific crosses.

### 4.11. Calculating Total Reproductive Isolation

The effect of a single barrier and the relative contribution to total reproductive isolation within both species pairs was calculated following the method of Sobel and Chen [61]:

(5) $${\mathrm{RI}}_{\mathrm{total}}=1-2\times \left(\frac{S\times H_{S}+U\times H_{U}}{S\times H_{S}+U\times {\;H}_{U}+S\times C_{S}+U\times C_{U}}\right)$$

where S refers to the extent of shared period of flowering, U refers to the unshared period of flowering, and H and C represent heterospecific and conspecific effects, respectively, but are multiplied across all components of RI and are considered both within the share (Hs, Cs) and the unshared (Hu, Cu) period of flowering (see [61]). To calculate the absolute contribution (AC) of a barrier, the combined isolation including the focal barrier and all preceding barriers was calculated using the equation of RI_total_. The calculation was then repeated, including all preceding barriers, but the focal barrier was excluded. The latter was subtracted from the former to reveal the absolute contribution of any individual barrier:

(6) $${\mathrm{AC}}_{i}={\mathrm{RI}}_{\left[1,\;i\right]}\;-{\mathrm{RI}}_{\left[1,\;i-1\right]\;}$$

where RI_[1, i]_ represents the combined isolation calculated by RI_total_ including all barriers from the first to act through the focal barrier (i). RI_[1, i−1]_ represents the same calculation excluding the focal barrier. The values of RI generally vary from −1 to 1, with −1 representing interspecific gene flow is facilitated, 1 representing a complete isolating barrier and 0 representing random mating between two species.

### 4.12. Statistical Analyses

In this study, all statistical analyses were done in R version 4.1.2 (R development Core Team, 2022), and data were expressed as the mean ± standard error. Differences in soil properties between the two *Salvia* species and among sites within species were compared using PERMANOVA, with the ‘adonis’ function of the vegan package of R [64] and with dissimilarity calculated as Bray–Curtis distances and 9999 permutations. We visualized differences in soil composition between *Salvia* species across different sites using nonmetric multidimensional scaling (NMDS). Independent sample *t*-tests were used to test for the significant differences in soil properties and floral traits between the two species. For each species, a Kruskal–Wallis nonparametric analysis of variance was used to compare the physical size of the most frequent visitors (i.e., *Bombus* species), floral constancy, and pollen–pistil interactions. We pooled data in 2015 and 2016 together to calculate floral constancy because the foraging bouts of some pollinators were not frequent. Means of floral constancy between years were compared with Wilcoxon paired signed rank tests to determine all pairwise differences for each analysis. A generalized linear model (GLM) with binomial errors was used to test the influence of hand pollination treatments and species on seed germination. Their interaction between the treatments and species was also included. The significance of the GLM models with likelihood-ratio tests was examined using a one-way ANOVA followed by a Tukey multiple comparison test using the glht function in the multcomp package [65].

## 5. Conclusions

Overall, our results showed strong but permeable reproductive isolation between subalpine populations of *S*. *digitaloides* and *S*. *flava*. The mechanical or ethological isolation through pollinator foraging behaviors, pollen–pistil interactions, and interspecific cross-pollinations reduced some degrees of interspecific gene exchange. However, they may not be sufficient to prevent hybridization as they are leaky. Both microhabitats and pollinators made substantial contributions to total reproductive isolation. Although microhabitat and pollinator differentiations prevented hybridization between the two species, the question remains regarding to what extent ecological selections associated with the habitat mosaic of the alpine vegetation account for the persistence of sympatric plant species. This question may be addressed by studying spatial patterns of soil microhabitats and their associations with environmental variables and effects on floral traits. Studying a larger range of species that includes the effectiveness of pollinators in pollen transfer between anthers and stigmas, pollen–pistil interactions at different flower life spans, and the impacts of nectar robbers on pollination and other pollinators may also allow us to estimate if prezygotic barriers are foremost in maintaining species integrity in these subalpine *Salvia* species.

Our study is unique in showing how soil microhabitat differentiation may cause floral trait differences between sympatric species, resulting in pollinator isolation by flower constancy. The present study highlighted the significant importance of post-pollination barriers to prevent gene flow between the two *Salvia* species and the importance of habitat heterogeneity to maintain species co-existence in a biodiversity hotspot.

## Figures and Tables

**Table 1:** Soil properties on the different study sites where *S*. *digitaloides* and *S*. *flava* occur.

Soil Properties	Sites	*S*. *digitaloides* (Mean ± SE)	*S*. *flava* (Mean ± SE)	*p* Value
	LABG	7.00 ± 0.16 ^c^	7.00 ± 0.15 ^b^	
Total potassium (K_2_O)(%)	WV	9.80 ± 0.51 ^b^	7.47 ± 0.49 ^b^	<0.01
	WL	15.07 ± 10.62 ^a^	9.07 ± 0.21 ^a^	
	LABG	6.00 ± 0.00 ^b^	5.40 ± 0.13	
pH	WV	6.13 ± 0.09 ^b^	5.73 ± 0.12	<0.001
	WL	6.33 ± 0.49 ^a^	5.47 ± 0.13	
	LABG	188.53 ± 9.28	184.40 ± 2.05 ^a^	
Silicon (g/kg)	WV	200.27 ± 5.78	142.40 ± 8.40 ^b^	<0.001
	WL	206.60 ± 198.47	179.20 ± 3.31 ^a^	
	LABG	18.13 ± 0.22 ^a^	13.20 ± 0.59 ^a^	
Titanium (g/kg)	WV	18.87 ± 0.36 ^a^	10.20 ± 1.03 ^b^	<0.001
	WL	17.13 ± 0.24 ^b^	14.73 ± 0.21 ^a^	
	LABG	56.40 ± 2.70 ^a^	259.33 ± 20.49 ^b^	
Soil organic matter (g/kg)	WV	60.73 ± 5.70 ^a^	399.467 ± 30.02 ^a^	<0.001
	WL	26.27 ± 2.34 ^b^	236.33 ± 14.91 ^b^	
	LABG	1.33 ± 0.13	12.00 ± 1.13 ^b^	
Total nitrogen (g/kg)	WV	1.33 ± 0.13	16.53 ± 1.30 ^a^	<0.001
	WL	1.07 ± 0.07	10.00 ± 0.58 ^b^	
	LABG	1.20 ± 0.22 ^a^	1.67 ± 0.29 ^a^	
Ammonium nitrogen (mg/kg)	WV	0.67 ± 0.21 ^b^	0.73 ± 0.21 ^b^	<0.065
	WL	0.20 ± 0.11 ^b^	0.73 ± 0.15 ^b^	
	LABG	1.2000 ± 0.17	3.33 ± 0.25	
Total phosphorus (P_2_O_5_)(%)	WV	1.13 ± 0.09	3.33 ± 0.13	<0.001
	WL	1.13 ± 0.09	3.20 ± 0.11	
	LABG	7.00 ± 0.95 ^a^	13.33 ± 1.33	
Available phosphorus (mg/kg)	WV	3.00 ± 0.58 ^b^	17.00 ± 1.96	<0.001
	WL	0.73 ± 0.12 ^c^	15.67 ± 1.65	
	LABG	177.27 ± 5.69 ^a^	346.67 ± 29.71	
Available potassium (mg/kg)	WV	209.07 ± 8.51 ^c^	348.33 ± 17.72	<0.001
	WL	69.00 ± 1.64 ^b^	322.00 ± 25.37	
	LABG	28.00 ± 0.13 ^a^	148.93 ± 12.31 ^b^	
Total carbon (mg/kg)	WV	1.33 ± 0.13 ^b^	233.20 ± 17.48 ^a^	<0.001
	WL	1.07 ± 0.07 ^c^	140.13 ± 8.27 ^b^	
	LABG	0.67 ± 0.13 ^b^	1.00 ± 0.00 ^a^	
Soil water content (%)	WV	1.00 ± 0.01 ^ab^	1.00 ± 0.01 ^a^	<0.230
	WL	0.40 ± 0.13 ^c^	0.40 ± 0.13 ^b^	
Nitrate-nitrogen (mg/kg)	LABG	23.33 ± 1.25 ^a^	51.53 ± 3.01	
	WV	7.93 ± 0.50 ^b^	48.87 ± 0.62	<0.001
	WL	7.60 ± 0.62 ^b^	51.80 ± 4.55	

Note: Different superscript letters indicate significant differences at *p* < 0.05 (Kruskal–Wallis test). LABG = Lijiang Alpine Botanical Garden, WV = Wenhai Village, and WL = Wenhai Lake.

**Table 2:** Microhabitat occurrence and co-occurrence of *Salvia digitaloides* and *S*. *flava* within 50 × 50 m quadrats at the study sites conducted in 2016 and 2019.

Year/Total	Overall Values of Sample Number	Number of Quadrats with *S. digitaloides* Only	Number of Quadrats with *S*. *flava* Only	Number of Quadrats with Both Species	RI Microhabitat for *S*. *digitaloides*	RI Microhabitat for *S*. *flava*
2016	140	60	73	7	0.90	0.91
2019	178	102	66	10	0.91	0.87
**Total**	**318**	**162**	**139**	**17**		

RI = Reproductive isolation.

**Table 3:** Floral trait measurements in *Salvia digitaloides* and *S*. *flava*.

Character	Unit (n)	*S*. *digitaloides* (Mean ± SE)	*S*. *flava* (Mean ± SE)	*t*	*p* Value
Inflorescence number per plant	n (30)	15.80 ± 1.18	5.33 ± 0.32	8.591	<0.001
Flower number per plant	n (30)	13.97 ± 1.31	5.30 ± 0.53	6.137	<0.001
Corolla tube length	mm (30)	33.71 ± 0.38	27.97 ± 0.24	12.809	<0.001
Corolla tube width	mm (30)	11.52 ± 0.27	11.02 ± 0.17	1.575	0.121
Stigma lever length	mm (30)	4.24 ± 0.10	7.25 ± 0.12	−19.941	<0.001
SLP	mm (30)	4.59 ± 0.31	7.81 ± 0.48	−4.994	<0.001
Style length	mm (30)	39.92 ± 0.38	33.21 ± 0.25	14.759	<0.001
SL	mm (30)	4.45 ± 0.23	9.93 ± 0.29	−14.985	<0.001
Opening length	mm (30)	7.50 ± 0.17	7.23 ± 0.12	1.304	0.197
Opening width	mm (30)	6.45 ± 0.19	5.16 ± 0.13	5.515	<0.001
Length of exerted stigma	mm (30)	3.36 ± 0.22	3.46 ± 0.29	−0.319	0.751
Lower lever’s arm height	mm (30)	2.88 ± 0.12	1.86 ± 0.13	5.712	<0.001
Flower longevity	day (30)	6.71 ± 0.28	4.83 ± 0.19	−5.443	<0.001
Nectar volume	µL (30)	11.81 ± 0.69	8.09 ± 0.93	3.163	<0.01
Sugar concentration	% (30)	22.47 ± 0.93	15.47 ± 1.45	4.063	<0.001
Pollen number	n (15)	43,356.00 ± 17,048.14	25,100.00 ± 3680.10	1.047	0.311
Pollen/ovule ratio	n (15)	10,839.00 ± 4262.04	6275.00 ± 920.02	1.047	0.311

SL = the distance between stigma and lever (horizontal); SLP = the distance between stigma and landing platforms.

**Table 4:** Intra- and inter-specific foraging bouts, degree of reproductive isolation (RI), and floral constancy (CI) for *S*. *digitaloides* (*SD*) and *S*. *flava* (*SF*).

		Number of Visited Flowers within and between the *Salvia* Species						
Pollinators	Interspecies/ Total Bouts	*SD* → *SD*	*SD* → *SF*	*SF* → *SF*	*SF* → *SD*	RI for *SD*	RI for *SF*	CI
*Bombus remotus*	0/35	-	-	218	-	-	1	1
*B. funararius*	7/40	-	4	365	3	−1	0.98	0.99 ± 0.001
*B. friseanus*	38/50	343	20	205	74	0.89	0.89	0.92 ± 0.05
*B. secures*	1/33	175	-	-	1	1	−1	1
*Macroglossum* species	1/16	67	1	53	-	0.97	1	1
*Dufourea carbopila*	1/14	36	-	-	1	1	−1	0.88 ± 0.09

Dashes (-) denote a solitary case where no pollinator visit was detected.

## Data Availability

The data that support the findings of this study are available from the corresponding authors upon reasonable request.

## References

[1] Coyne J., Orr H. (1998). The evolutionary genetics of speciation. Philos. Trans. R. Soc..

[2] Coyne J.A. (2007). Sympatric speciation. Curr. Biol..

[3] Nosil P. (2012). Ecological Speciation.

[4] Coyne J.A., Orr H.A. (2004). Speciation.

[5] Schluter D., Conte G.L. (2009). Genetics and ecological speciation. Proc. Natl. Acad. Sci. USA.

[6] Huber F.K., Kaiser R., Sauter W., Schiestl F.P. (2005). Floral scent emission and pollinator attraction in two species of *Gymnadenia* (Orchidaceae). Oecologia.

[7] Sweigart A.L., Fishman L., Willis J.H. (2006). A simple genetic incompatibility causes hybrid male sterility in *Mimulus*. Genetics.

[8] Eaton D.A.R., Fenster C.B., Hereford J., Huang S.Q., Ree R.H. (2012). Floral diversity and community structure in *Pedicularis* (Orobanchaceae). Ecology.

[9] Esposito F., Vereecken N.J., Gammella M., Rinaldi R., Laurent P., Tytea D. (2018). Characterization of sympatric *Platanthera bifolia* and *Platanthera chlorantha* (Orchidaceae) populations with intermediate plants. PeerJ.

[10] Aguilar-Rodríguez P.A., Tschapka M., García-Franco J.G., Krömer T., MacSwiney M.C. (2019). Bromeliads going batty: Pollinator partitioning among sympatric chiropterophilous Bromeliaceae. AoB Plants.

[11] Paudel B.R., Burd M., Shrestha M., Dyer A.G., Li Q.J. (2018). Reproductive isolation in alpine gingers: How do coexisting *Roscoea* (*R*. *purpurea* and *R*. *tumjensis*) conserve species integrity. Evolution.

[12] Tao Z.B., Ren Z.X., Bernhard P., Liang H., Li H.D., Zhao Y.H., Wang H., Li D.Z. (2018). Does reproductive isolation reflect the segregation of color forms in *Spiranthes sinensis* (Pers.) *Ames complex* (Orchidaceae) in the Chinese Himalayas?. Ecol. Evol..

[13] Schouppe D., Brys R., Vallejo-Marin M., Jacquemyn H. (2017). Geographic variation in floral traits and the capacity of autonomous selfing across allopatric and sympatric populations of two closely related *Centaurium* species. Sci. Rep..

[14] Butlin R.K., Galindo J., Grahame J.W. (2008). Sympatric, parapatric or allopatric: The most important way to classify speciation?. Philos. Trans. R. Soc. B Biol. Sci..

[15] Zhao J.L., Gugger P.F., Xia Y.M., Li Q.J. (2001). Ecological divergence of two closely related *Roscoea* species associated with late Quaternary climate change. J. Biogeogr..

[16] Martíneza J.J., Anzeiger V.D.C. (2011). Geographic distribution and phenetic skull variation in two close species of *Graomys* (Rodentia, Cricetidae, Sigmodontinae). Zool. Anz..

[17] Grant V. (1981). Plant Speciation.

[18] Hodges S.A., Arnold M.L. (1994). Floral and ecological isolation between *Aquilegia formosa* and *Aquilegia pubescens*. Proc. Natl. Acad. Sci. USA.

[19] Husband B.C., Sabara H. (2003). Reproductive isolation between autotetraploids and their diploid progenitors in fireweed, Chamerion angustifolium (Onagraceae). New Phytol..

[20] Yang C.F., Gituru R.W., Guo Y.H. (2007). Reproductive isolation of two sympatric louseworts, *Pedicularis rhinanthoides* and *Pedicularis longiflora* (Orobanchaceae): How does the same pollinator type avoid interspecific pollen transfer?. Biol. J. Linn. Soc..

[21] Ma Y.P., Zhou R., Milne R. (2016). Strong reproductive isolation despite occasional hybridization between a widely distributed and a narrow endemic *Rhododendron* species. Front. Plant Sci..

[22] Pascarella J.B. (2007). Mechanisms of prezygotic reproductive isolation between two sympatric species, *Gelsemium rankinii* and *G*. *sempervirens* (Gelsemiaceae), in the Southeastern United States. Am. J. Bot..

[23] Bibi F., Tomlinson K., Liu C.G., Liu C.A.L., Jin Y.Q., Tang J.W. (2022). Fine root production and soil available nutrients in rubber monoculture versus rubber-*Flemingia macrophylla* agroforestry. Forests.

[24] Kay K.M. (2006). Reproductive isolation between two closely related hummingbird-pollinated neotropical gingers. Evolution.

[25] Zhang H., Tao Z., Trunschke J., Shrestha M. (2022). Reproductive isolation among three nocturnal moth-pollinated sympatric *Habenaria* species (Orchidaceae). Front. Plant Sci..

[26] Whitehead M.R., Peakall R. (2014). Pollinator specificity drives strong prepollination reproductive isolation in sympatric sexually deceptive orchids. Evolution.

[27] Hopkins R., Rausher M.D. (2012). Pollinator-mediated selection on flower color allele drives reinforcement. Science.

[28] Ramsey J., Bradshaw H.D., Schemske D.W. (2003). Components of reproductive isolation between the monkey flowers *Mimulus lewisii* and *M*. *cardinalis* (Phrymaceae). Evolution.

[29] Dell’Olivo A., Hoballah M.E., Gübitz T., Kuhlemeier C. (2011). Isolation barriers between *Petunia axillaris* and *Petunia integrifolia* (Solanaceae). Evolution.

[30] Liang H., Ren Z.X., Tao Z.B., Zhao Y.H., Bernhard P., Li D.Z., Wang H. (2018). Impact of pre- and post-pollination barriers on pollen transfer and reproductive isolation among three sympatric *Pedicularis* (Orobanchaceae) species. Plant Biol..

[31] Grant V. (1994). Modes and origins of mechanical and ethological isolation in angiosperms. Proc. Natl. Acad. Sci. USA.

[32] Yogan M., Bernard P., Christian R., Aude C.C., Catherine F. (2020). Pollination ecology, specialization, and genetic isolation in sympatric bee-pollinated *Salvia* (Lamiaceae). Int. J. Plant Sci..

[33] Claßen-Bockhoff R., Speck T., Tweraser E., Wester P., Thimm S., Reith M. (2004). The staminal lever mechanism in *Salvia* L. (Lamiaceae): A key innovation for adaptive radiation?. Org. Divers. Evol..

[34] Wester P., Claßen-Bockhoff R. (2011). Pollination syndromes of new world *Salvia* species with special reference to bird pollination. Ann. Missouri Bot. Gard..

[35] Cuevas E., Espino J., Marques I. (2018). Reproductive isolation between *Salvia elegans* and *S*. *fulgens*, two hummingbird-pollinated sympatric sages. Plant Biol..

[36] Wei Y., Huang Y., Li G. (2017). Reproductive isolation in sympatric *Salvia* species sharing a sole pollinator. Biodivers. Sci..

[37] Grant K.A., Grant V. (1964). Mechanical isolation of *Salvia apiana* and *Salvia mellifera* (Labiatae). Evolution.

[38] Wester P., Claßen-Bockhoff R. (2007). Floral diversity and pollen transfer mechanisms in bird-pollinated *Salvia* species. Ann. Bot..

[39] Zhang B., Li Q.J. (2014). Phenotypic selection on the staminal lever mechanism in *Salvia digitaloides* (Labiaceae). Evol. Ecol..

[40] Dai W., Yang Y., Patch H.M., Christina M., Grozinger J.M. (2022). Soil moisture affects plant-pollinator interactions in an annual flowering plant. Philos. Trans. R. Soc. B Biol. Sci..

[41] Osborne J.L., Martin A.P., Carreck N.L., Swain J.L., Knight M.E., Goulson D., Hale R.J., Sanderson R.A. (2008). Bumblebee flight distances in relation to the forage landscape. J. Anim. Ecol..

[42] Rao S., Strange J.P. (2012). Bumble bee (Hymenoptera: Apidae) foraging distance and colony density associated with a late-season mass flowering crop. Environ. Entomol..

[43] Wang Z.Y., Ge Y.X., Scott M., Spangenberg G. (2004). Viability and longevity of pollen from transgenic and nontransgenic tall fescue (*Festuca arundinacea*) (Poaceae) plants. Am. J. Bot..

[44] Ackerman J.D., Carromero W. (2005). Is reproductive success related to color polymorphism in a deception pollinated tropical terrestrial orchid?. Caribb. J. Sci..

[45] Arida B.L., Scopece G., Machado R.M., Moraes A.P., Forni-Martins E., Pinheiro F. (2021). Reproductive barriers and fertility of two neotropical orchid species and their natural hybrid. Evol. Ecol..

[46] Paudel B.R., Shrestha M., Dyer A.G., Zhu X.F., Abdusalam A., Li Q.J. (2015). Out of Africa: Evidence of the obligate mutualism between long corolla tubed plant and long-tongued fly in the Himalayas. Ecol. Evol..

[47] Zhang Y.W., Zhao J.M., Inouye D.W. (2014). Nectar thieves influence reproductive fitness by altering behaviour of nectar robbers and legitimate pollinators in *Corydalis ambigua* (Fumariaceae). J. Ecol..

[48] Rojas-Nossa S.V., Sánchez J.M., Navarro L. (2006). Effects of nectar robbing on male and female reproductive success of a pollinator-dependent plant. Ann. Bot..

[49] Richman S.K., Barker J.L., Baek M., Papaj D.R., Irwin R.E., Bronstein J.L. (2021). The sensory and cognitive ecology of nectar robbing. Front. Ecol. Evol..

[50] Ye X.M., Jin X.F., Nouye D.W., Wang Q.F., Yang C.F. (2018). Variation in composition of two bumble bee species across communities affects nectar robbing but maintains pollinator visitation rate to an alpine plant, *Salvia przewalskii*. Ecol. Entomol..

[51] Ott D., Hühn P., Claßen-Bockhoff R. (2015). *Salvia apiana*—A carpenter bee flower?. Flora Morphol. Distrib. Funct. Ecol. Plants.

[52] Tong Z.Y., Huang S.Q. (2016). Pre- and post-pollination interaction between six co-flowering *Pedicularis* species via heterospecific pollen transfer. New Phytol..

[53] Liang H., Zhao Y.H., Rafferty N.E., Ren Z.X., Zhong L., Li H.D., Li D.Z., Wang H. (2021). Evolutionary and ecological factors structure a plant-bumblebee network in a biodiversity hotspot, the Himalaya-Hengduan Mountains. Funct. Ecol..

[54] Brach A.R., Song H. (2006). eFloras: New directions for online floras exemplified by the flora of China project. Taxon.

[55] Zhang B., Sun S., Fang Q.E., Bai X.M. (2013). Evolutionary response of staminal lever mechanism of different species in *Salvia* to spatial variation in pollinators. Chin. J. Plant Ecol..

[56] Hu G.X., Takano A., Drew B.T., Liu E.-D., Soltis D.E., Soltis P.S., Hua Peng H., Xiang C.L. (2018). Phylogeny and staminal evolution of *Salvia* (Lamiaceae, Nepetoideae) in East Asia. Ann. Bot..

[57] Sun R., Lan G., Yang C., Wu Z., Chen B. (2021). Effects of tropical rainforest conversion to rubber plantation on soil quality in Hainan Island, China. Biogeosciences.

[58] Chen C., Liu W., Wu J., Jiang X., Zhu X. (2019). Can intercropping with the cash crop help improve the soil physico-chemical properties of rubber plantations?. Geoderma.

[59] Zhang J., Zhang J.L., Zhou L.L., Ma N.M., Jia Y.F., Yang F., Zhou H.Y., Cao X.Y. (2019). Influence of soil moisture content and soil and water conservation measures on time to runoff initiation under different rainfall intensities. Catena.

[60] Deng Y.S., Xia D., Cai C.F., Ding S.W. (2016). Effects of land uses on soil physio-chemical properties and erodibility in collapsing-gully alluvial fan of Anxi County, China. J. Integr. Agric..

[61] Sobel J.M., Chen G.F. (2014). Unification of methods for estimating the strength of reproductive isolation. Evolution.

[62] Gegear R.J., Thomson J.D. (2004). Does the flower constancy of bumble bees reflect foraging economics?. Ethology.

[63] Vance N.C., Bernhardt P., Edens R.M. (2004). Pollination and seed production in *Xerophyllum tenax* (Melanthiaceae in the Cascade Range of central Oregon. Am. J. Bot..

[64] Oksanen J., Blanchet F.G., Kindt R., Legendre P., Minchin P.R., O’Hara R.B., Simpson G.L., Solymos P., Stevens M.H.H., Wagner H. (2014). Vegan: Community Ecology Package. R Package Version 2.2-0. http://CRAN.Rproject.org/package=vegan.

[65] Hothorn T., Bretz F., Westfall P. (2008). Simultaneous inference in general parametric models. Biom. J..

